# Early Growth and Protein-Energy Metabolism in Chicken Lines Divergently Selected on Ultimate pH

**DOI:** 10.3389/fphys.2021.643580

**Published:** 2021-03-04

**Authors:** Sonia Métayer-Coustard, Sophie Tesseraud, Christophe Praud, David Royer, Thierry Bordeau, Edouard Coudert, Estelle Cailleau-Audouin, Estelle Godet, Joël Delaveau, Elisabeth Le Bihan-Duval, Cécile Berri

**Affiliations:** ^1^INRAE, Université de Tours, BOA, Nouzilly, France; ^2^INRAE, PEAT, Nouzilly, France

**Keywords:** chicks, metabolism, muscle, protein synthesis, glycogen, carbohydrate metabolism

## Abstract

In chickens, a divergent selection on the *Pectoralis major* pHu allowed the creation of the pHu+ and pHu− lines, which represent a unique model for studying the biological control of carbohydrate storage in muscle. The present study aimed to describe the early mechanisms involved in the establishment of pHu+ and pHu− phenotypes. At hatching, pHu+ chicks were slightly heavier but exhibited lower plasma glucose and triglyceride and higher uric acid. After 5 days, pHu+ chicks exhibited higher breast meat yield compared to pHu− while their body weight was no different. At both ages, *in vivo* muscle glycogen content was lower in pHu+ than in pHu− muscles. The lower ability of pHu+ chicks to store carbohydrate in their muscle was associated with the increased expression of *SLC2A1* and *SLC2A3* genes coding glucose transporters 1 and 3, and of *CS* and *LDHα* coding key enzymes of oxidative and glycolytic pathways, respectively. Reduced muscle glycogen content at hatching of the pHu+ was concomitant with higher activation by phosphorylation of S6 kinase 1/ribosomal protein S6 pathway, known to activate protein synthesis in chicken muscle. In conclusion, differences observed in muscle at slaughter age in the pHu+ and pHu− lines are already present at hatching. They are associated with several changes related to both carbohydrate and protein metabolism, which are likely to affect their ability to use eggs or exogenous nutrients for muscle growth or energy storage.

## Introduction

To answer the growing demand for poultry meat, breeding companies have focused their efforts on improving live performances (i.e., growth rate and feed efficiency) and breast meat yield (Rauw et al., 1998; Havenstein et al., 2003a,b). The selection of meat-type chicken lines for increased growth and muscle development has been accompanied by significant anatomical, physiological, and metabolic changes. As a result, limitations appear in terms of physiology affecting both animal robustness and product quality. In these meat-type chicken lines, a decrease in muscle energy reserves, evaluated through *in vivo* glycogen content, is observed (Berri et al., 2001, 2007). In chicken, there is a strong genetic link (with a correlation of −0.97) between the muscle glycogen reserves available *in vivo* and the ultimate pH (pHu) of the meat (Le Bihan Duval et al., 2008). Changes in pHu affect most of the meat quality traits such as color, water-holding capacity, texture, and processing ability (Le Bihan-Duval et al., 2008; Petracci et al., 2015). Recent studies also highlighted high pHu as a predisposing factor favoring the emergence of white striping (WS) and wooden breast (WB) defects in heavy broiler lines (Kuttappan et al., 2009; Petracci et al., 2013; Abasht et al., 2016; Alnahhas et al., 2016). Several studies have shown that chicken breast meat pHu is a highly heritable trait that can therefore be modified by genetic selection (Le Bihan Duval et al., 2001, 2008). On this basis, a divergent selection on breast meat pHu was initiated from a common fast-growing grand-parental population in order to increase (in the high pHu line, pHu+) or decrease (in the low pHu line, pHu−) the genetic value for pHu. After six generations of divergent selection, the two divergent lines exhibited a difference of around 0.5 pH unit, that was associated with significant differences in muscle glycogen and sensory and technological meat quality (Alnahhas et al., 2014, 2015). It also appeared that while body weight at 6 weeks was similar between the two lines, pHu+ birds exhibited higher thigh and breast muscle yield than pHu− birds. As already described for heavy broiler lines, the incidence of WS was higher in pHu+, the line presenting higher pHu and lower muscle glycogen reserves (Alnahhas et al., 2016). On the other hand, neither the pHu− nor the pHu+ line is affected by the WB syndrome. The etiology of WS and WB is poorly understood, but a new hypothesis suggests a potential dysregulation of lipid and glucose metabolism in these muscle disorders (Lake and Abasht, 2020). Regarding metabolism, the depletion of glycogen reserves that characterizes the pHu+ line is associated with a partial reorientation of muscle metabolism towards oxidative stress, protein catabolism, and lipid beta-oxidation rather than glycolysis, as well as an over-activation of processes related to muscle remodeling (Beauclercq et al., 2016, 2017). Conversely, carbohydrate metabolites are over-represented in the serum and muscle of the pHu− birds, consistent with their high level of muscle glycogen content. The purpose of the present study was to determine the extent to which the phenotypic differences between lines were programmed early, by considering chicks at the time of hatching or after a few days of nutrition. To improve our understanding of the molecular mechanisms involved in the establishment of line-related phenotypic differences, several molecular actors related to energy metabolism and protein synthesis were explored *in vivo* and *in vitro*.

## Materials and Methods

### Animals and Muscle Tissue Collection

All investigators were certified by the French government to carry out animal experiments. All procedures were approved by the French Agricultural Agency and the Scientific Research Agency (under referenced authorization N° 201,503,111,710,750). This study was conducted in accordance with the guidelines for Care and Use of Agricultural Animals in Agricultural Research and Teaching. The study used fast-growing chicks belonging to the eighth generation of selection for high (pHu+) or low (pHu−) *Pectoralis major* muscle pHu. Thirty chicks (males and females) from both lines were hatched and sacrificed by cervical dislocation (D0 that corresponds to the time when chicks were taken out from the hatchery). A second batch of thirty chicks per line (males and females) were reared for 5 days under conventional conditions at PEAT INRAE Poultry Experimental Facility (2018, https://doi.org/10.15454/1.5572326250887292E12), as described by Alnahhas et al. (2014). Males and females of both divergent lines were reared as a single population. They were fed and watered *ad libitum* until 8 h before sacrifice by cervical dislocation at D5. A third batch of birds from both lines (425 in total) was also reared until slaughter age (42 days) following classical rearing conditions (Alnahhas et al., 2014).

Blood samples were collected at day 0 (D0) and day 5 (D5) on heparin (135 USP U Lithium Heparin, VENOSAFE, Dominique Dutscher, Brumath, France), stored on ice, centrifuged at 3000 *g* for 10 min at 4°C, and plasma aliquoted and frozen at −80°C until analysis. Tissues were sampled from hatched chicks (D0; liver, *Pectoralis major*, and pipping muscles) and 5-day-old chicks (D5; liver and *Pectoralis major*) of both lines. The pipping muscle is a cervical muscle, which is solicited for shell breaking and regresses after hatching (Pulikanti et al., 2010). At each age of sampling (D0 and D5), both muscle types and the liver of eight females and eight males per line were sampled, weighed, and frozen in liquid nitrogen before storage at −80°C until further molecular and biochemical analyses. At D0 and D5, *Pectoralis major* muscles from six male chicks of each line were snap-frozen in isopentane cooled in liquid nitrogen before storage at −80°C until further histochemical study. Fresh muscle samples were finally kept for 24 h for pHu measurement.

### Cell Culture

The primary cell culture studies were conducted as previously described (Praud et al., 2017) in which procedures were validated to study muscle biology in the chicken model. Briefly, the *Pectoralis major* muscles were sliced with a scalpel and washed with sterile PBS for 15 min. After 1 min of gentle centrifugation (20 *g*) and supernatant discarding, myogenic cells derived from satellite cells were extracted by enzymatic dissociation with 0.14% pronase (Sigma Aldrich, Lyon, France) for 10 min at room temperature. After centrifugation (20 *g*, 30 s, at room temperature), the supernatant was kept while the pellet was treated three times more with pronase. All supernatants were gathered and centrifuged at 200 *g* for 10 min at room temperature. Pelleted cells were then suspended in a DMEM-HAMF12 culture medium containing 3 g/L glucose (Sigma Aldrich, Lyon, France) and supplemented with 10% of fetal calf serum (Sigma Aldrich, Lyon, France), 2% ultroser G (PALL biosepra, Cergy, France) containing penicillin and streptomycin 1%, and antifungal fungizone (Sigma-Aldrich, Lyon, France).

The cells were seeded in six-well plates at 30,000 cells/cm^2^ in a complete DMEM-HAMF12 proliferation medium (DMEM-HamF12 with 10% fetal calf serum, 2% of ultroser G, and 0.365 g/L glutamine) for cell stimulation experiments. After 48 h of proliferation, the cell culture medium was replaced by a DMEM-HAMF12 differentiation medium, containing the same antibiotics and antifungal as the proliferation medium, but supplemented with 2% horse serum and 0.5% fetal calf serum. After 48 h of differentiation, the cells were washed once with PBS and weaned in DMEM-HAMF12 serum-free medium for 4 h. Then, cells were incubated in a DMEM-HAMF12 medium supplemented or not with insulin (100 nM) for 0, 15, or 45 min to measure the response of protein synthesis pathways to insulin. This stimulation was stopped by washing with cold PBS and cells were recovered by scraping with 200 μl of lysis buffer (composition). The cell extracts were then lysed for 30 min at 4°C before centrifugation (12,000 *g*, 30 min, 4°C) and supernatants were sampled and stored at −80°C until further SDS-PAGE and Western blot analyses.

### Immunocytochemistry

Immunocytochemistry procedures were done on cells cultured in plates and fixed using cold methanol, as previously described by Praud et al. (2017). After blocking with goat serum 10% (Sigma), the cells were incubated with primary antibodies against sarcomeric myosin (MF20, 1/50) for 1 h. The MF20 antibody developed by Donald A. Fischman was obtained from the Developmental Studies Hybridoma Bank, created by the NICHD of the NIH and maintained at The University of Iowa, Department of Biology, Iowa City, IA 52242. The immunolocalization was performed using a biotinylated goat anti-mouse antibody (anti IgG H+L, Southern Biotech) and with streptavidin-Cy2 (Southern Biotech). All nuclei were labeled using DAPI 0.05 μg/ml (Sigma). Fluorescence imaging on cells was done using a Zeiss Axiovert 200 Microscope (Carl Zeiss S.A.S) and a Zeiss Camera AxioCam MRm (Carl Zeiss S.A.S) controlled by the Axiovision software (Axiovision Rel. 4.6.3; Carl Zeiss Imaging Solutions, GmbH, 2007).

### Histochemical Traits

Ten-micrometer-thick *Pectoralis major* muscle cross-sections were used to perform periodic acid-Schiff (PAS) staining. They were fixed in Carnoy’s fixative and incubated for 5 min in periodic acid 1% and 90 min in Schiff’s reagent (Sigma-Aldrich, Saint-Quentin Fallavier, France) before washing with distilled water, dehydration, and mounting in Canada balsam (Sigma Aldrich, Saint-Quentin Fallavier, France).

### Plasma Metabolite Measurements

Glycemia, uric acid, and triglyceride were measured using commercial kits (RTUTM Glucose, Enrique Uric Acid PAP 150, Enzyme Triglycerides PAP 150, Biomérieux, Craponne, France). The free amino acids (Free AA) were measured after extraction with 10% (v/v) of sulfosalicylic acid using 2% of ninhydrin reagent (Sigma Aldrich, Lyon, France). L-serine was used as a standard (Dupont et al., 2008). All enzymatic colorimetric assays were performed in 96-well plates using a TECAN infinite M200 spectrophotometer according to the supplier’s recommendations (Artiss and Entwistle, 1981; Fossati and Prencipe, 1982).

### Liver and Muscle Glycogen Content

About 500 mg of frozen *Pectoralis major*, pipping muscle, and liver sampled within 10 min post-mortem was powdered and used to estimate the glycogen content. Tissue *in vivo* glycogen content was estimated through the measurement of the glycolytic potential that reflects the glycogen that is in the tissue prior to slaughter as it considers the main intermediates of glycogen degradation (glucose-6-phosphate, free glucose, and lactate; Monin and Sellier, 1985). Glycogen was measured through enzymatic procedures according to Dalrymple and Hamm (1973). The glycolytic potential (GP) was calculated according to the equation: GP = 2[glycogen + glucose + glucose-6-phosphate] + lactate, and was expressed as μmoles of lactate equivalent per gram of tissue.

### Ultimate pH Measurement in *Pectoralis major* Muscle

For D0 and D5 samples, after storage (24 h at 4°C), 1 g of *Pectoralis major* muscle was crushed with an Ultra-Turrax T25 (Janke and Kunkelika-labortechnick) in 9 ml of a solution of KCl-iodoacetate (sodium iodoacetate 5 mM, KCl 150 mM). The pH of this solution was then measured using a portable pH meter (model 506; Crison Instruments SA, Alella, Bercelona, Spain) with a glass electrode.

At day 42, pHu was recorded in the right *Pectoralis major* muscles sampled on chickens originating from 213 birds from the pHu+ line and 212 birds from the pHu− line. At this age, the pHu was measured 24 h post-mortem using a portable pH meter (model 506, Crison Instruments SA, Alella, Barcelona, Spain) by direct insertion of its glass electrode into the muscles.

### WS Incidence

The prevalence and severity of WS were assessed on the right *Pectoralis major* muscle using the scoring grid of Kuttappan et al. (2012). Muscles were classified as normal (WS0) in the absence of WS, moderate (WS1) when the thickness of the white stripes was less than 1 mm, or severely affected (WS2) when the thickness of the stripes was equal to or greater than 1 mm.

### Western Blotting and Cell Signaling

Tissue lysates were prepared for cell signaling analyses as previously described in Duchêne et al. (2008). Lysates were first centrifuged at 1000 *g* for 30 min at 10°C and supernatants were then centrifuged at 31,000 *g* for 45 min at 10°C. Solubilized lysates (containing 40 μg of protein) were subjected to SDS-PAGE and Western blotting with the appropriate antibody. Antibodies raised against phospho-S6K1 [T389], phospho-S6 [S235/S236], and S6 ribosomal protein, were obtained from Cell Signaling Technology (Beverly, MA, United States). Anti-S6K1, and anti-vinculin antibodies were obtained from Santa Cruz Biotechnology (Santa Cruz, CA, United States), Upstate (Millipore, Paris, France), and Sigma Chemical Company (St. Louis, MO, United States).

After washing, membranes were incubated with a DyLight® 680-conjugated antibody. Alexa Fluor 680-conjugated secondary antibodies were obtained from Molecular Probes (Invitrogen, Carlsbad, CA, United States). Bands were visualized with Infrared Fluorescence using the Odyssey® Imaging System (LI-COR Inc. Biotechnology, Lincoln, NE) and quantified with the Odyssey imaging system software (Application software, version 1.2).

### RNA Isolation and RT-qPCR

Total RNA was extracted from 100 mg of tissue samples using RNA Now (Biogentec, Seabrook, TX, United States) according to the manufacturer’s recommendations. After RNase-Free DNase treatment (Ambion, Clinisciences, Montrouge, France), RNA was reverse-transcribed using Super Script II RNase H Reverse Transcriptase (Invitrogen, Carlsbad, CA, United States) with Random Primers (Promega, Charbonnieres-les-Bains, France). The sequences of forward and reverse primers used to amplify chicken *SLC2A1* (**NM_205209.1**), *SLC2A3* (**NC_006088.5**), *SLC2A8* (**NM_204375.1**), *SLC2A12* (**XM_419733.4**), β-hydroxyacyl-CoA dehydrogenase (*HAD*; **NM_001277897.1**), citrate synthase (*CS*; **XM_015300289.1**), lactate dehydrogenase (*LDH*; **XM_015864180**), and housekeeping *18S rRNA* (**AF173612.1**) genes were specifically designed or reproduced from the literature (Dupont et al., 2008; Joubert et al., 2010; Coudert et al., 2015, 2018); these are presented in Table 1. Each cDNA sample was amplified in triplicate by real-time PCR using Sybr Green I Master kit (Roche, Mannheim, Germany) on a LightCycler® 480 II apparatus (Roche, Meylan, France). Gene expression levels were estimated based on PCR efficiency and threshold cycle (Ct) deviation of an unknown sample vs. a control, as previously described (Pfaffl, 2001). The expression of the studied genes was normalized with the *18S rRNA* housekeeping gene that was confirmed to be invariable.

**Table 1:** Sequences of the forward and reverse primers used to quantify gene expression by real-time PCR.

Genes^1^	Forward	Reverse
*18S*	TCC AGC TAG GAA TAA TGG AAT AGG A	CCG GCC GTC CCT CTT AAT
*CS*	AGG GAT TTC ATC TGG AAC ACA CT	CAC CGT GTA GTA CTT CAT CTC CT
*HAD*	ATC CTT GCA AAT CAC GCA GTT	AAT GGA GGC CAC CAA ATC G
*LDH*	TTA ACT TGG TCC AAC GCA ACG TCA AT	TCC ACT GGG TTT GAG ACA ATC AG
*SLC2A1*	ACA ACA CCG GCG TCA TCA A	TTG ACA TCA GCA TGG AGT TAC G
*SLC2A3*	TGC TCA TCT TCT TCA TAT TCA CAT	ACT TCT TTG TCA GGT TCT ATG C
*SLC2A8*	CTG GAG GAA TAC TGG GAG GC	CAC CAC CAT CAA CTG GAC AA
*SLC2A12*	AGA GAG TGG GGA GGT TCC C	TCA GGA CGA GCC AAG ACA

1 *18S*, 18S ribosomal RNA (used as housekeeping gene); *CS*, citrate synthase; *HAD*, β-hydroxyacyl-CoA dehydrogenase; *LDH*, lactate dehydrogenase; *SLC2A1*, solute carrier family 2 member 1; *SLC2A3*, solute carrier family 2 member 3; *SLC2A8*, solute carrier family 2 member 8; *SLC2A12*, solute carrier family 2 member 12.

### Statistics

Values are presented as mean ± SEM. Results were analyzed firstly by three-way ANOVA (including sex, line, and age effect) after having checked the normality of the residual distribution and the homoscedasticity. When there was no sex effect, the results were analyzed by one- or two-way ANOVA, to test the line effect or line × age effect, respectively. The accepted type I error was set at 5%. Comparisons of means for each significant effect were performed using Fisher’s least significant difference test. When the residuals were not normally distributed and variances were not homogeneous between groups, data were analyzed with the non-parametric Kruskal-Wallis test and a multiple comparison test. Differences were considered to be significant when *p* values were below 0.05.

## Results

### Body Weight and Composition

There was no sex effect on the parameters of body weight and composition (data not shown). The pHu+ chicks exhibited a slightly higher (+3.9%, *p* = 0.01) average body weight at hatching (D0) than pHu− chicks, but their liver and *Pectoralis major* muscle weights and yields were similar (Table 2). The difference in body weight between the two lines was no longer significant 5 days post-hatching, while the *Pectoralis major* muscle weight and yield of pHu+ chicks became 10% (*p* = 0.005) and 8% (*p* = 0.0006) higher than those of pHu− chicks, respectively (Table 2).

**Table 2:** Body composition of chicks of the pHu+ and pHu− lines at hatching (D0) and 5 days post-hatching (D5).

Age	Lines	Body weight (g)	PM^1^ weight (g)	Liver weight (g)	PM yield^2^	Liver yield^2^
D0	pHu+	42.4 ± 0.46	0.52 ± 0.02	0.98 ± 0.02	1.21 ± 0.03	2.30 ± 0.05
	pHu−	40.8 ± 0.41	0.48 ± 0.01	0.94 ± 0.02	1.27 ± 0.03	2.26 ± 0.05
	Line effect^3^	*P* = 0.01	NS	NS	NS	NS
D5	pHu+	99.0 ± 1.40	4.39 ± 0.09	3.27 ± 0.09	4.43 ± 0.07	3.29 ± 0.06
	pHu−	96.0 ± 1.97	3.92 ± 0.13	3.05 ± 0.12	4.07 ± 0.08	3.17 ± 0.08
	Line effect^3^	NS	0.005	NS	0.0006	NS

Data are presented as mean ± SEM (*n* = 30 per line).

1 PM = *Pectoralis major* muscle.

2 % of body weight.

3 NS = not significant.

### Plasma Metabolites

**Figure 1:**
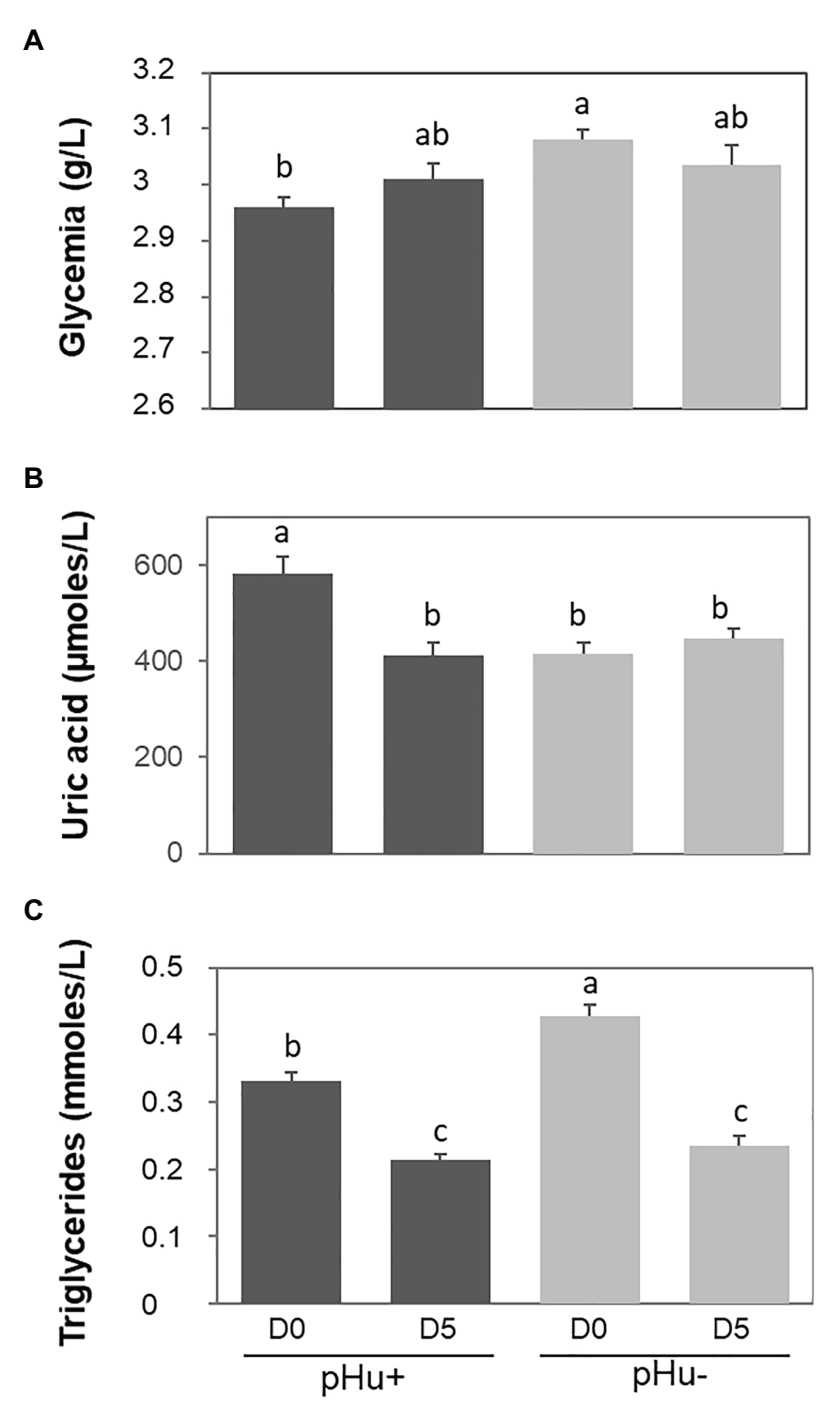
Plasma concentration in glucose (glycemia, **A**), uric acid **(B)**, and triglyceride **(C)** measured at hatching (D0) and 5 days post-hatching (D5) in the pHu+ and pHu− lines. Data are presented as mean ± SEM; *n* = 15 per line and per age. Different letters indicate significant differences between muscle groups (*p* ≤ 0.05).

Plasma glucose and triglyceride concentrations were higher in pHu− hatched chicks while uric acid concentrations were higher in pHu+ ones (Figure 1). There was no effect of the line on free AA and no sex effect on all the characters measured at the plasma level (data not shown). Free AA concentration was no different between D0 and D5. Uric acid concentrations significantly decreased from D0 to D5 in pHu+ birds (−37%, *p* < 0.0001) while they remained constant in pHu− birds (Figure 1). Uric acid concentrations were in turn similar between the two lines at D5. On the other hand, triglyceride concentrations decreased in both lines between the time of hatching and D5 at the rate of −35 and −46% (*p* < 0.0001) in pHu+ and pHu−, respectively (Figure 1). As for uric acid concentrations, no differences were observed for triglycerides between the two lines at D5, as was also the case for plasma glucose.

### Muscle and Liver Characteristics

**Figure 2:**
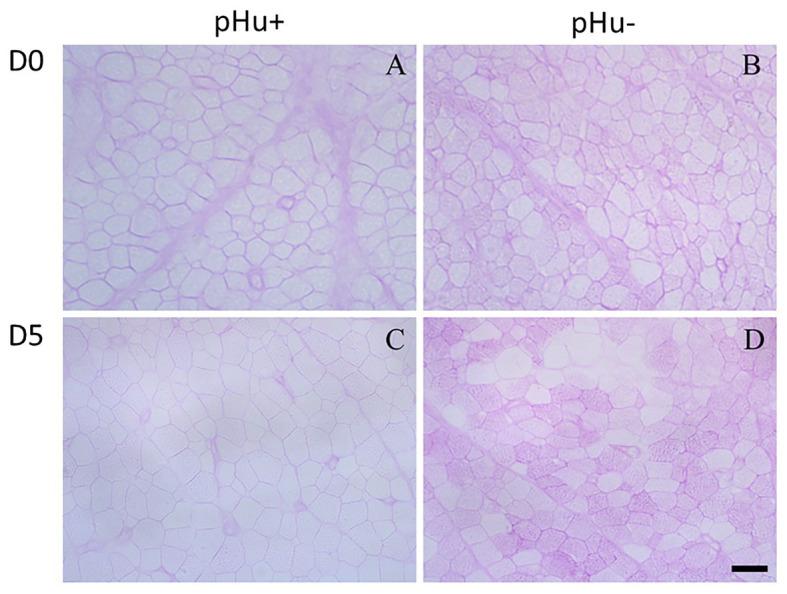
Periodic acid-Schiff staining of *Pectoralis major* muscle cross-sections from pHu+ (**A,C)** vs. pHu− **(B,D)** at hatching (D0, **A,B**) and 5 days post-hatching (D5, **C,D**). Scale bar = 13 μm **(A,B)** and 25 μm **(C,D)**.

**Figure 3:**
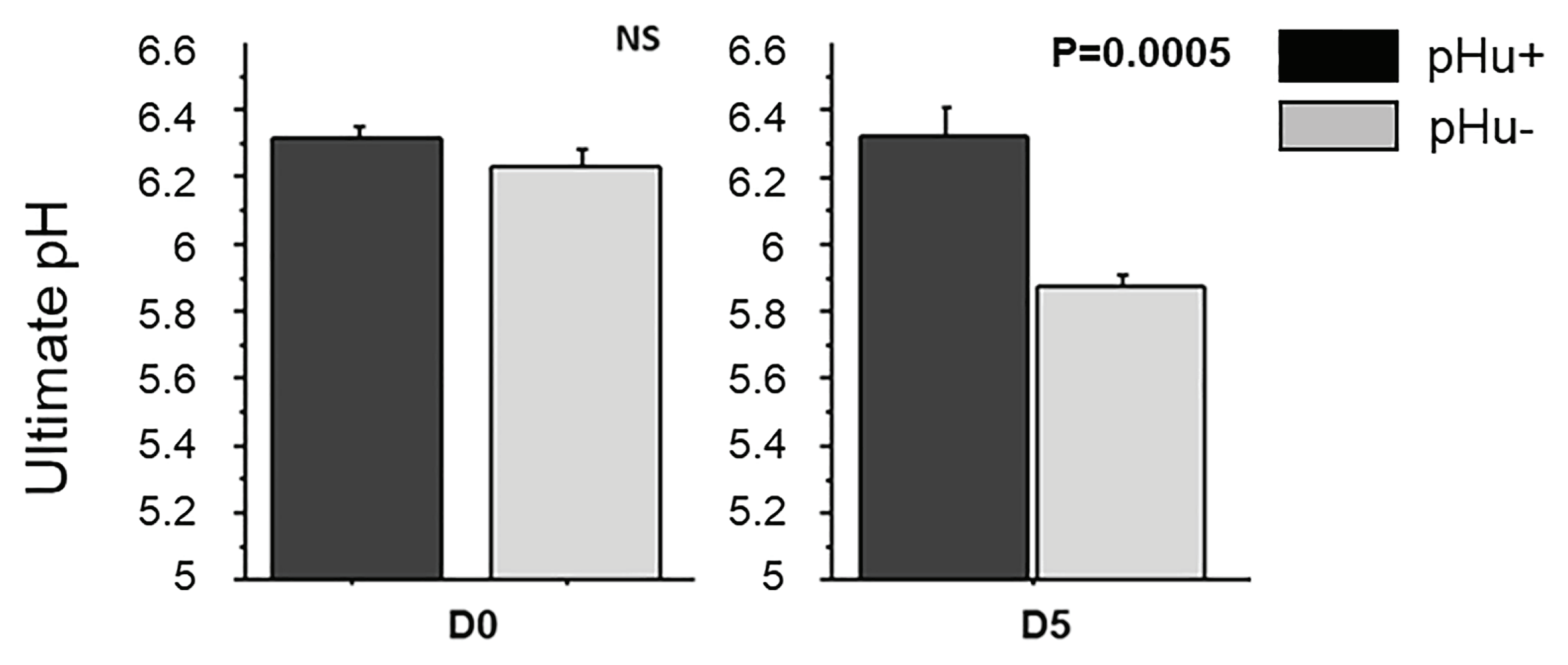
Ultimate pH in *Pectoralis major* muscles at hatching (D0) and at 5 days post-hatching (D5) in the pHu+ and pHu− lines. Data are presented as mean ± SEM; *n* = 6 per line and per age at D0 and D5.

There was no difference between the lines in the lactate and glycogen contents in the liver of hatched and 5-day-old chicks (Table 3). At hatching, glycogen content was higher in pHu− muscles, with differences being more pronounced in the pipping than in the *Pectoralis major* muscle (Table 3). Higher values of glycogen were also observed 5 days post-hatch in the *Pectoralis major* muscle of pHu− compared to pHu+ chicks. In the pipping muscle, which is highly solicited during the hatching process, the lactate content measured around 10 min post-mortem was also higher in pHu− than in pHu+ hatch chicks. Higher values of lactate were also observed in the *Pectoralis major* muscle of pHu− compared to pHu+ chicks 5 days post-hatching (Table 3). Regardless of the line, both lactate and glycogen contents increased between D0 and D5 in the *Pectoralis major* muscle (*p* < 0.0001; tested by two-way ANOVA, including age and line as the main effects). Differences in *Pectoralis major* muscle fiber glycogen content between the lines were clearly observable by microscopy after PAS staining at both ages (Figure 2). Despite the differences in *in vivo* glycogen content observed at hatching between the pHu + and pHu− lines, their pHu (measured 24 h post-mortem) was no different (Figure 3). By contrast, a difference in pHu of 0.45 units was observed 5 days after hatching between pHu+ and pHu− chicks.

**Table 3:** Lactate and glycogen contents in the muscle and liver of the pHu+ and pHu− lines at hatching (D0) and 5 days post-hatching.

Age	Tissue	Metabolites^1^	pHu+	pHu−	Line effect^2^
D0	Liver	Lactate	13.66 ± 0.15	13.79 ± 0.11	NS
		Glycogen content	116.97 ± 4.76	105.60 ± 6.77	NS
	*PM* muscle^3^	Lactate	24.48 ± 1.24	25.90 ± 1.37	NS
		Glycogen content	108.50 ± 4.71	122.87 ± 4.61	0.04
	Pipping muscle	Lactate	23.40 ± 0.85	31.71 ± 1.96	0.001
		Glycogen content	111.40 ± 3.81	142.49 ± 6.68	0.0008
D5	Liver	Lactate	14.51 ± 0.20	14.89 ± 0.25	NS
		Glycogen content	90.83 ± 3.45	95.74 ± 3.66	NS
	*PM* muscle	Lactate	37.88 ± 1.11	42.47 ± 1.45	0.02
		Glycogen content	119.80 ± 2.94	149.78 ± 4.14	<0.0001

Data are presented as mean ± SEM (*n* = 8 males and *n* = 8 females per line).

1 Lactate expressed as μmol/g; glycolytic potential expressed as μmol equivalent lactate/g.

2 NS = not significant.

3 *PM* muscle = *Pectoralis major* muscle.

At slaughter age (42 days), the pHu of the *Pectoralis major* muscle was very different between lines: 6.12 vs. 5.54 (*p* < 0.001) in the pHu+ and pHu− lines, respectively. Similarly, the incidence and severity of the WS defect was very different between pHu+ and pHu− lines (*p* < 0.001). Thus, the proportion of unaffected pectoral muscles (WS0) was 61.3% in the pHu− while it was only 38.5% in the pHu+ line. Conversely, the proportions of moderately (WS1) or severely (WS2) affected muscles were higher in the pHu+ (41.8 and 19.7%, respectively) than in the pHu− line (31.1 and 7.6%, respectively). Whether for pHu or the WS defect, we did not observe any effect of sex on these traits (data not shown).

### Gene Expression in *Pectoralis major* Muscles

The mRNA expression levels of genes coding the main isoforms of the glucose transporter (GLUT), i.e., *SLC2A1*, *SLC2A3*, *SLC2A8*, and *SLC2A12*, were measured in the *Pectoralis major* of the two lines at hatching and 5 days later (Table 4). There was no sex effect on the expression of these genes (data not shown). There was an age × line interaction for the *SLC2A12* expression. The *SLC2A12* expression increased between hatching and D5 while that of *SLC2A1*, *SLC2A3*, and *SLC2A8* decreased. Among these genes, only the expressions of *SLC2A1* and *SLC2A3* were upregulated in pHu+ compared to pHu− muscles, whether at D0 or D5.

**Table 4:** Relative mRNA abundance of genes^1^ coding *SLC2A1*, *SLC2A3*, *SLC2A8*, *SLC2A12*, *CS*, *LDHa*, and *HAD* in the *Pectoralis major* muscles of chicks of the pHu+ and pHu− lines measured at hatching (D0) and 5 days post-hatching (D5).

Age	D0		D5		Effect^2^		
Lines	pHu+	pHu−	pHu+	pHu−	Age	Line	Age × line
*SLC2A1*	1.04 ± 0.08	0.91 ± 0.05	0.87 ± 0.06	0.66 ± 0.04	<0.0001	0.0005	NS
*SLC2A3*	1.17 ± 0.08	1.03 ± 0.04	0.82 ± 0.04	0.69 ± 0.02	<0.0001	0.01	NS
*SLC2A8*	1.18 ± 0.08	1.30 ± 0.07	0.55 ± 0.03	0.47 ± 0.03	<0.0001	NS	NS
*SLC2A12*	0.36 ± 0.03^a^	0.42 ± 0.02^a^	1.14 ± 0.07^b^	1.00 ± 0.06^b^	<0.0001	NS	0.03
*CS*	1.26 ± 0.08	1.14 ± 0.05	0.75 ± 0.03	0.61 ± 0.02	<0.0001	0.0008	NS
*HAD*	0.92 ± 0.07	0.82 ± 0.05	0.90 ± 0.05	0.76 ± 0.03	0.08	NS	NS
*LDHa*	0.15 ± 0.02	0.11 ± 0.01	1.78 ± 0.1	1.54 ± 0.09	<0.0001	0.02	NS
*LDHa/CS*	0.08 ± 0.01	0.09 ± 0.01	2.68 ± 0.09	2.81 ± 0.14	<0.0001	NS	NS

Data are presented as mean ± SEM (*n* = 8 males and *n* = 8 females per line). 18S was used for normalization.

1 *CS*, citrate synthase; *HAD*, β-hydroxyacyl-CoA dehydrogenase; *LDH*, lactate dehydrogenase; *SLC2A1*, solute carrier family 2 member 1; *SLC2A3*, solute carrier family 2 member 3; *SLC2A8*, solute carrier family 2 member 8; *SLC2A12*, solute carrier family 2 member 12.

2 NS = not significant.

a,b Within a row, mean values without a common letter differ between groups (*p* ≤ 0.05).

The mRNA expression of genes coding key enzymes of glycolytic and oxidative metabolisms, i.e., citrate synthase (CS), 3-hydroxyacyl-CoA-dehydrogenase (HAD), and lactate dehydrogenase (LDH*α*), were also measured in the *Pectoralis major* muscle of the two lines (Table 4). There were no age × line interactions in any of them. The CS expression almost halved between hatching and day 5, while LDHα expression strongly increased (up to 10 times). The HAD expression remained quite stable during this period. The CS and LDHα expressions were greater in the pHu+ muscle. The calculation of the ratio LDH/CS (glycolysis with pyruvate reduction/aerobic potential of the Krebs cycle) has been proposed by Hochachka et al. (1983) to classify muscles according to their glycolytic capacity. LDH/CS ratio, and therefore glycolytic capacity, significantly increased between D0 and D5 but no differences were observed between the pHu+ and pHu− lines.

### Myogenic Differentiation in pHu+ and pHu− Chicks

**Figure 4:**
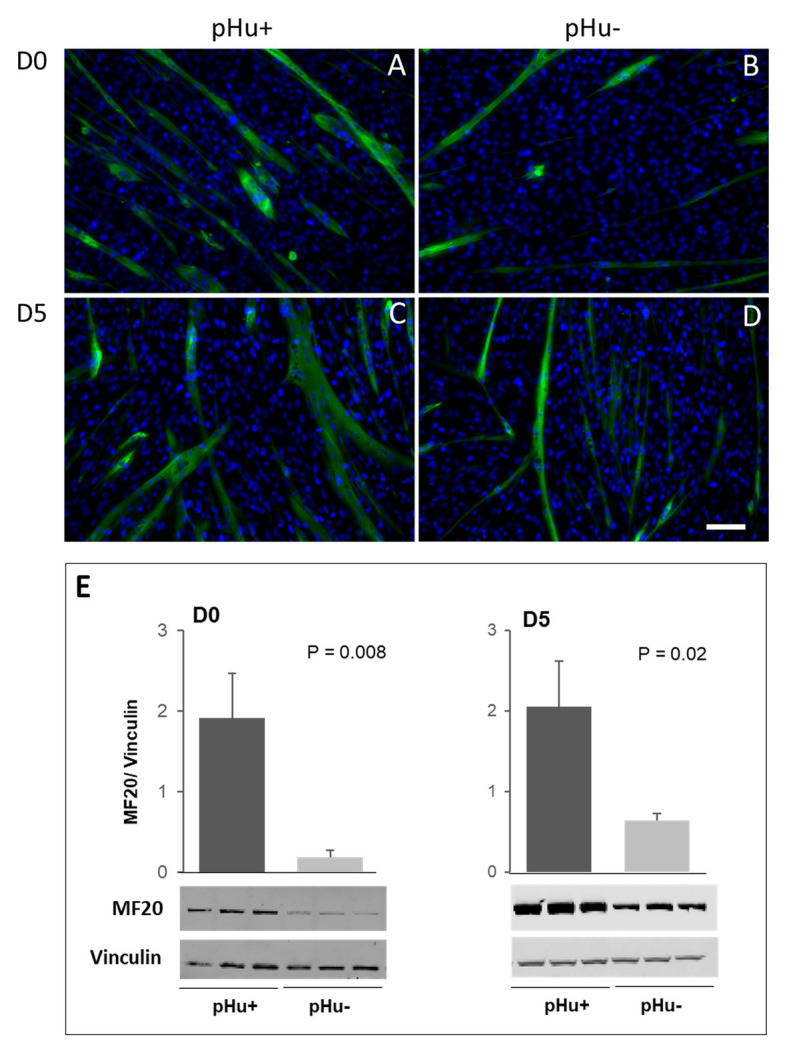
Immunolocalization **(A–D)** and quantification **(E)** of sarcomeric myosin (MF20) on satellite cells derived from *Pectoralis major* muscles sampled at hatching (D0) and 5 days post-hatching (D5) in the pHu+ and pHu− lines. Scale bar = 70 μm. Data are expressed as mean ± SEM. Different letters indicate significant differences between muscle groups (*p* ≤ 0.05). The anti-vinculin antibody was used as a loading control.

The immunolocalization and quantification of the sarcomeric myosin (MF20) were assessed *in vitro* to compare the differentiation ability of myogenic cells from pHu+ and pHu− lines. Immunolocalization of MF20 was done on primary cell cultures prepared from pHu+ or from pHu− *Pectoralis major* muscles at D0 and D5. Myoblasts derived from pHu+ showed a better ability to differentiate (Figures 4A,C) compared to pHu−-derived myoblasts (Figures 4B,D). This was confirmed by Western blot, which revealed a higher expression of the MF20 protein in pHu+ compared to pHu− cell lysates, regardless of sampling age (Figure 4E).

### S6K1 and S6 Activation

**Figure 5:**
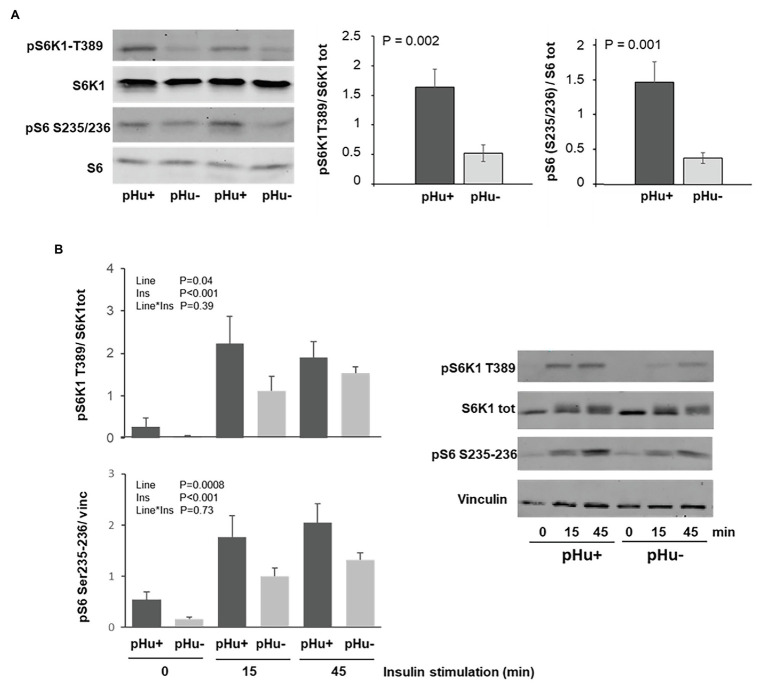
Regulation of the S6K1 pathway in the *Pectoralis major* muscles sampled in the pHu+ and pHu− at hatch (D0; **A**) and in the cell culture of satellite cells from *Pectoralis major* muscles sampled at hatching (D0) following insulin stimulation (100 nM for 0, 15, or 45 min; **B**). Membranes were incubated with polyclonal antibodies against phospho-S6K1 [T389] (pS6K1 T389), S6K1, phospho-S6 [S235/236] (pS6 S235/236), and ribosomal protein S6. Vinculin was used as loading control. Data are expressed as mean ± SEM.

The ribosomal proteins S6 kinase 1 (S6K1) and ribosomal protein S6 (S6) are key regulators of muscle cell growth and protein synthesis. Their activation by phosphorylation, on threonine 389 for S6K1 and on serine 235/236 for S6, was measured in *Pectoralis major* muscles at hatching (D0) both *in vivo* and *in vitro*. The phosphorylation *in vivo* of both S6K1 and S6 was three times higher in the *Pectoralis major* muscles of the pHu+ line than those of the pHu− line at hatching (Figure 5A). No sex effect was observed upon activation of the S6K1/ribosomal protein S6 pathway. Greater phosphorylation of S6K1 and S6 was also observed *in vitro* in a primary cell culture prepared from pHu+ than from pHu− *Pectoralis major* muscles, whether under basal conditions or following insulin stimulation (muscles sampled at hatching, Figure 5B). Similarly, the S6K1/ribosomal protein S6 pathway was more activated in myotubes originating from pHu+ *Pectoralis major* muscles in chicks at D5, than in those from the pHu− line (data not shown).

## Discussion

In chickens, a near-perfect genetic correlation (of −0.97) was established between the *in vivo* glycogen content of breast muscle and pHu, which is a key factor of meat quality (Le Bihan-Duval et al., 2008). In this species, selection for increased growth rate and/or breast muscle mass has been associated with reduced *in vivo* glycogen storage and in turn with higher pHu (Berri et al., 2001, 2007); according to recent studies, this could be a condition associated with a greater occurrence of degenerative disorders such as WS and WB defects (Kuttappan et al., 2009; Petracci et al., 2013; Abasht et al., 2016; Alnahhas et al., 2016, 2017). The understanding of the biological determinism of glycogen content is thus a key point for the control of muscle integrity and meat quality in chickens. While several studies have studied these processes at slaughter age in relation to the control of meat quality and muscle integrity, none have focused on the earlier stages of chicken development. The two broiler lines divergently selected for breast muscle pHu constitute a unique resource population to explore the molecular pathways involved in the control of muscle glycogen storage. To add to the studies carried out at slaughter age (Beauclercq et al., 2016, 2017), this study aims to investigate both *in vivo* and *in vitro* whether phenotypic divergence is established earlier during development, by considering pHu+ and pHu− chicks at hatching, before any exogenous nutrient intake, and after 5 days on a starting diet composed mainly of carbohydrates.

According to our results, pHu+ chicks are heavier at hatching than pHu− chicks. In chickens, the hatching weight is highly related to egg weight (Halbersleben and Mussehl, 1922; Iqbal et al., 2017). Although we did not measure this trait in the studied generation (G8), we have since acquired data that show a significantly higher egg weight in the pHu+ compared to the pHu− line; e.g., in the 9th generation, the pHu+ egg weight was 57.9 ± 0.3 g compared to 54.7 ± 0.2 g for the pHu− line (*p* < 0.0001). We also clearly showed that differences in muscle glycogen exist as soon as hatching occurs, with pHu− chicks exhibiting higher glycogen reserves in both the *Pectoralis major* muscle, targeted by the selection on the ultimate pH of the meat, and the pipping muscle, which is highly solicited at hatching for shell breaking. This result is consistent with previous observations, which showed that the selection on breast meat pHu was affected in the same way but to a lower extent of glycogen storage in thigh *Sartorius* muscle (Alnahhas et al., 2014). By contrast, selection on muscle pHu did not alter liver glycogen, suggesting a specific effect of selection on skeletal muscle energy stores. In the egg, less than 1% of the total available nutrients are carbohydrates and only 0.3% are free glucose. Thus, glucose homeostasis during late embryonic development depends on the amount of glucose generated by gluconeogenesis from amino acids originating from the degradation of proteins (including muscle proteins) and which are stored as glycogen in the liver. The glycerol issued from the triglyceride hepatic metabolism is the main substrate for liver and muscle glycogen synthesis at the end of incubation (Sunny and Bequette, 2011). Between embryonic days 15 and 19, liver metabolism is activated mainly to ensure the transfer of glucose and fatty acids to muscles solicited for hatching, such as the pipping muscle whose contents in glucose, glycogen, and proteins increase during this period (Pulikanti et al., 2010). During the late-hatching stage, carbohydrates rather than lipids are preferentially metabolized, and as a consequence, glycogen is highly consumed. This induces a high rate of muscle protein degradation and of amino acid production for gluconeogenesis (Chen et al., 2009; Noy and Uni, 2010). This intensive use of proteins for an energy purpose at the end of embryogenesis specially affects *Pectoralis major* muscle development, whose yield relative to the weight of the embryo decreases between embryonic day 11 and hatching (Guernec et al., 2003). Accordingly, an increase in the expression of atrophy-related genes involved in muscle protein degradation such as atrogin-1 and MuRF1 is observed in *Pectoralis major* muscles between embryonic day 18 and hatching, which likely contributes to the decreased rate of breast muscle development (Everaert et al., 2013). Our study revealed higher blood uric acid contents in the hatched pHu+ chicks, which likely indicates a higher rate of muscle protein catabolism and amino acid utilization to produce energy in a context of muscle glycogen depletion, compared to pHu− chicks. Moreover, even though pHu+ chicks were heavier at hatching than pHu− ones, the two lines exhibited similar *Pectoralis muscle* weights and yields. At hatching, pHu− chicks exhibited higher glycemia, which was already observed at 6 weeks of age (Beauclercq et al., 2015), and higher blood triglyceride contents than pHu+ chicks, indicating that metabolic differences exist between the two lines independently of any exogenous nutrient supply. Higher blood glucose and triglyceride contents in pHu− may reflect a better use of yolk lipid as source of energy during embryogenesis, in agreement with the greater occurrence of highly resorbed yolk observed at hatching in this line compared to the pHu+ one (data not shown).

Obviously, differences in glucose homeostasis exist between the lines before hatching, when lipids are the principal source of nutrients for the embryo. However, the higher glycemia of the pHu− chicks may also reflect lower glucose uptake by muscle cells compared to pHu+ chicks. Similar to mammals, the striated skeletal muscle in birds is a major glucose-utilizing tissue. Glucose is an essential metabolic substrate that is transported into cells by several glucose transporters belonging to the GLUT family. As for mammals, glucose transport in chickens involves several glucose transporters that play a crucial role in cellular glucose uptake and glucose homeostasis. Among them, GLUT1, 3, 8, and 12 are expressed in chicken muscles (Kono et al., 2005; Coudert et al., 2015, 2018; Shimamoto et al., 2016), with the levels of mRNA expressions of GLUT1, 3, and 8 (but not GLUT12) being particularly high during the post-hatching period in the glycolytic *Pectoralis major* muscle compared to more oxidative muscles (Shimamoto et al., 2016). Moreover, although GLUT4 is the major glucose transporter in mammalian muscles, it is not expressed in chickens and the insulin-sensitive glucose transporter GLUT12 could partially play the role of GLUT4 in this species (Coudert et al., 2015, 2018). In our study, both *SLC2A1* and *SLC2A3* genes, coding, respectively, for GLUT1 and 3, were significantly more expressed in the pHu+ *Pectoralis major* muscles compared to those of pHu− chicks, both at hatching and at day 5. The mRNA expressions of *SLC2A1* have been shown to gradually decrease during late embryogenesis (from day 12 to 18) before increasing again between hatching and 5 days post-hatching in chicken *Pectoralis major* muscles (Coudert et al., 2018), when breast muscle growth rate and therefore energy requirement is maximal. The higher expressions of *SLC2A1* and *SLC2A3* in the *Pectoralis major* of pHu+ chicks through the enhancement of glucose uptake from blood into muscle could therefore contribute to the lower glycemia of pHu+ at hatching. Upon hatching, pHu+ muscles also exhibited lower glycogen stores compared to pHu− muscles. It could be hypothesized that despite a possibly higher glucose uptake in pHu+ muscles, the consumption of glucose for ATP production is also more intense than in pHu−. These results, obtained in the early stages of development in chicks, may be of more general interest, especially concerning the later appearance of degenerative muscle disorders in fast-growing chickens, which, according to several authors, would imply dysregulation of carbohydrate metabolism (for review, see Lake and Abasht, 2020).

A greater glucose uptake capacity has already been associated with greater metabolic demand when comparing muscles from chicken lines selected for high or low body weights (Zhang et al., 2013). It is likely that the pHu+ muscles exhibited a higher metabolic demand than pHu− muscles, since they are characterized by a greater activation of the S6K1/ribosomal protein S6 signaling pathway that is involved in the stimulation of growth and protein synthesis. The higher propensity of pHu+ chicks for muscle protein synthesis at hatching was observed both *in vitro*, with greater phosphorylation of S6K1 and ribosomal protein S6 in primary muscle cells with or without insulin stimulation, and *in vivo* in the *Pectoralis major* muscle sampled at hatching. This higher protein synthesis in the pHu+ muscles of hatched chicks is likely to be accompanied by higher muscle protein degradation to provide amino acid for energy production during the late-hatching stage. Moreover, higher proteolysis and AA catabolism has already been observed in the breast muscles of pHu+ compared to pHu− chickens at slaughter age (Beauclercq et al., 2016). Altogether, our findings suggest that breast muscle deposition could require a more intense protein turnover in pHu+ around hatching, likely leading to a higher energy requirement compared to pHu−, since both protein degradation and synthesis are energetically costly processes. This is consistent with the higher expression of genes coding LDH and CS, which are indicative of the glycolytic and oxidative capacities, respectively, and lower glycogen contents in the *Pectoralis major* muscles of pHu+ compared to pHu− lines.

Between hatching and 5 days post-hatching, blood triglycerides decreased in the two lines, likely in response to dietary carbohydrate instead of the lipids and AA mainly provided *in ovo* (Moran, 2007; Cherian, 2015). After 5 days, there was no further line effect on uric acid, triglyceride, or glycemia. However, at day 5, the pHu+ chicks exhibited higher *Pectoralis major* muscle weights and yields compared to pHu− chicks. As shown by the strong increase in LDH/CS ratio, there is a shift from oxidative to glycolytic metabolism between hatching and day 5 in the breast muscles of both lines (Hochachka et al., 1983). Both lines exhibited similar LDH/CS ratios, suggesting that they have similar metabolic profiles at this age despite the higher metabolic activity and lower glycogen content observed in pHu+ muscles.

The pHu+ muscles of 5-day-old chicks were also characterized by a greater expression of both *SLC2A1* and *SLC2A3* genes coding for glucose transporters 1 and 3, respectively. This could indicate that between hatching and day 5, the glucose transport is greater in pHu+ muscles both to face the lack of carbohydrate availability and to support the higher breast muscle growth rate observed post-hatching in this line compared to the pHu− one.

*In vitro* experiments clearly showed the higher capability of myoblasts derived from pHu+ muscles to differentiate in myotubes compared to those derived from pHu− ones, which is concomitant with the higher activation of the S6K1/S6 pathway in muscle cells derived from the pHu+ line, a pathway known to stimulate protein synthesis in chickens as in other species (Tesseraud et al., 2006; Nakashima et al., 2020). As mentioned above, the better ability of pHu+ muscles to activate the cascading phosphorylation of the S6K1/S6 pathway was also shown *in vivo* at hatching. Therefore, both *in vitro* and *in vivo* results indicated the greater propensity of pHu+ chicks for growth and muscle protein synthesis compared to pHu− ones. As discussed earlier, a higher protein synthesis rate of pHu+ chicks accompanied by higher muscle catabolism for energy supply during the late incubation phase may explain why pectoral muscle deposition was similar between pHu+ and pHu− lines at hatching. By contrast, during early post-hatching growth when chicks received carbohydrates and proteins as main sources of nutrients, a higher protein synthesis rate of pHu+ clearly led to higher muscle deposition compared to pHu−, while differences in glycogen content increased between the two lines. Breast muscle glycogen content was around 20% lower in pHu+ than in pHu− 5 days post-hatching, while it was less than 12% lower at hatching. According to the present study and our previous observations, the differences observed 5 days post-hatching remained until slaughter age (i.e., 6 weeks), when pHu+ chickens exhibited higher breast and thigh muscle yields and lower muscle glycogen, and thus higher pHu, compared to pHu− chickens (Alnahhas et al., 2014, 2015; present study). Such a competition between muscle protein and energy deposition had already been suggested in other fast-growing strains of chickens. Indeed, the selection for growth and breast meat yield in chickens led to decreased muscle glycogen content, affecting meat pHu and related quality traits, such as color and water-holding capacity (Berri et al., 2001, 2007).

In conclusion, this study had provided new information regarding the early development of phenotypic differences in two divergent chicken lines on the ultimate pH of breast meat, as well as the mechanisms involved. It revealed that muscle metabolic differences, in particular the glycogen content, induced by the selection at slaughter age, are already present at hatching and amplified by the exogenous supply of nutrients during the early post-hatching stage. Interestingly, reduced muscle glycogen content at hatching appeared to be associated with enhanced glucose transport and a higher propensity of muscle cells for protein synthesis, leading to greater breast muscle growth after hatching. However, the question of whether the higher protein turnover associated with the low carbohydrate reserves that characterize the pHu+ line from hatching onwards may contribute to their greater propensity to develop WS-type defects at slaughter age requires further study. All metabolic differences observed at hatching between the two lines are likely to be related to differences in nutrient use or availability *in ovo*, which deserves further investigation to decipher how embryos from the two lines interact with the main sources of nutrients during incubation, i.e., the yolk sac and amniotic fluid, and to determine if part of the effects of selection on the ultimate pH of breast meat are due to changes in their composition.

## Data Availability Statement

The raw data supporting the conclusions of this article will be made available by the authors, without undue reservation.

## Ethics Statement

The animal study was reviewed and approved by the French Agricultural Agency and the Scientific Research Agency.

## Author Contributions

Conceived and designed the experiments: SM-C, CB, and EB-D. Performed the experiments: DR, TB, EC, EC-A, EG, CP, JD, and SM-C. Analyzed the data: SM-C, CP, and EC-A. Wrote the paper: SM-C under the supervision of CB, ST, and EB-D. All authors contributed to the article and approved the submitted version.

### Conflict of Interest

The authors declare that the research was conducted in the absence of any commercial or financial relationships that could be construed as a potential conflict of interest.
